# Parents’ Experiences and Perspectives of Their Child’s Sleep Quality During Hospitalization

**DOI:** 10.1177/00099228231188223

**Published:** 2023-07-26

**Authors:** Cor-Jan van der Perk, Pia Burger, Jolanda Maaskant, Reinoud J. B. J. Gemke

**Affiliations:** 1 Emma Children’s Hospital, Amsterdam UMC, University of Amsterdam, Amsterdam, The Netherlands; 2 Department of Internal Medicine, Amsterdam UMC, University of Amsterdam, Amsterdam, The Netherlands

**Keywords:** sleep quality, child, parents, hospital, disrupters

## Abstract

Sleep is essential for maintenance and restoration of health, yet studies exploring this in hospitalized children are scarce. In a qualitative study, we assessed the perceived quality of sleep, factors affecting sleep, and the role of health care professionals in the sleep environment for hospitalized children aged 1 to 12 years. Data were obtained from 11 semi-structured, audio-recorded, and verbatim-transcribed interviews with parents, and analyzed using a systematic thematic analysis. The interviews were coded based on iterative assessment of transcripts. Subsequently, categories and interpretative main themes were identified. Four themes emerged: (1) being informed, keeping informed; (2) coordination of care; (3) parents as main advocates for their child’s sleep; and (4) environmental disturbers. Parents reported differences in their child’s sleep quality during hospital compared with home. Sleep is substantially affected during hospitalization, prompting the need for interventions to improve the quality of sleep of children. Parents provided valuable suggestions for improvements.

## Introduction

Sleep is an essential daily requirement for the development and maintenance of physical and psychological health such as memory processing and consolidation, cellular repair, brain development, and hormonal regulation.^1-5^ Good sleep quality is characterized by adequate sleep duration, adequate sleep quality (comprising sleep satisfaction, uninterrupted sleep, and being fit upon awakening), and adequate timing (a sleep schedule that fits with the natural biological rhythm, in the absence of sleep disorders).^6,7^

Reduced sleep quality is associated with unfavorable consequences on health and daily life. Short-term consequences of interrupted sleep are, for example, increased stress, emotional distress and mood disorders, and decreased cognition and memory.^8,9^ Adolescents show problems with school performance and have increased risk-taking behaviors.^9^ In addition, children are at risk of daytime sleepiness, reduced alertness, hyperactivity and decreased attention, and poor emotion regulation.^10,11^ Long-term health consequences of interrupted sleep in adults include anxiety, depression, hypertension, cardiovascular disease, weight-related issues, and type 2 diabetes mellitus.^8-10^

Particularly during hospitalization, the quality of sleep is important because sleep deprivation may contribute to impaired recovery, prolonged length of stay, reduced subjective well-being, and poor patient perception of hospitalized care.^12-14^ Nevertheless, research showed that sleep quality in hospitalized children is reduced compared with home.^15-18^

Studies exploring the experiences regarding children’s sleep quality during hospitalization are scarce. A qualitative study with hospitalized adolescents (13-17 years) revealed that a lack of self-control, disease-related factors, and unfamiliar noises affected sleep.^19^ One study reported on the views of parents about the sleep quality of their child (3-12 years) during hospitalization but did not include surgical patients. Results showed that noise, light, and daily schedules affected sleep.^20^ Another study focused on pediatric nurses’ views on factors affecting children’s sleep during hospitalization and their most important finding was that most influencing factors were related to nursing practice and could be resolved through changed policies and mentorship aimed at improving sleep for pediatric patients.^21^

To the best of our knowledge, no study on this subject has been executed among a general pediatric hospital population. Therefore, the objective of this study was to gain information on (1) the perceived quality of sleep, (2) disrupters and promoters of sleep, and (3) the role of health care professionals in creating a healthy sleep environment for children aged 1 to 12 years during hospitalization, as reported by parents.

## Methods

### Study Design

A deductive explorative qualitative study design with semi-structured interviews was used. We considered a phenomenological approach, the appropriate method for answering our research question, as it focuses on shared experiences and meanings among the participants using interviews about experiences and feelings.^22^ The COnsolidated criteria for REporting Qualitative research (COREQ) was used to ensure the accuracy of the design and execution of the study.^23^

### Setting and Participants

The study was executed at Emma Children’s Hospital, Amsterdam University Medical Center, 1 of the 7 tertiary care hospitals for children in the Netherlands. In this hospital, children from birth to 18 years old are treated on 3 general pediatric wards, namely, an acute admission ward, a pediatric intensive care unit, and a neonatal intensive care unit. Children stay in single or double patient rooms, accompanied by one of their parents. All rooms have sleeping facilities (a couch that can be converted into a bed) for parents and are equipped with toilets and showers. Every room has blackout curtains and a tablet on which relaxing music or lullabies can be played. Pedagogical supportive care provides age-related materials and care (eg, relaxing exercises, music mobiles) to help the children relax and potentially facilitate sleep. Children are also encouraged to bring their own items from home to support sleep.

Parents of children admitted on 2 general pediatric wards and the acute admission ward were invited to participate in the study. The fourth ward focuses on the care for children younger than 1 year old and therefore did not participate. The following inclusion criteria were used: parents of children aged 1 to 12 years rooming-in for at least one night, children staying at the ward for at least 24 hours, and parents’ proficiency in Dutch. For more unequivocal results, infants have been omitted due to unstable circadian rhythms. Children with known sleep disorders were excluded from the study. The participants were selected purposively. To reach maximum heterogeneity, we selected children with various disorders, ages, and length of stay. In addition, diversity in age and sex of parents were considered.^24^

### Data Collection

Eligibility for participation was assessed by C.J.v.d.P. in collaboration with the pediatric nurses. If parents were willing to participate, the interview was scheduled to take place during admission.

The interviews were conducted by C.J.v.d.P., who was not involved in the care for the patients. Characteristics of children and parents (eg, sex parent/patient, age, marital status, length of hospital stay, number of previous hospitalizations, reason for admission) were retrieved at the beginning of the interview. Interviews took place in a meeting room or patients’ room between October 2021 and March 2022, and lasted between 45 and 60 minutes. The interviews were audio recorded and field notes were written down immediately after each interview.

The structure of the interviews was guided by a topic list and a semi-structured interview guide, based on 2 studies.^19,25^ The guide was assembled in collaboration with the research team. After 2 interviews, the interview data were reviewed by C.J.v.d.P. and P.B. to determine whether adjustments in the interview guide were necessary. After updating the guide, the following interviews were executed.

Data saturation was determined in a research meeting in which all members agreed that no additional information could be derived from the interviews. Table 1 displays the topic list; the complete interview guide is shown in Supplemental Appendix A.

**Table 1.** Topic List.

Topic	Parents perceptions on
Usual sleep at home	Usual sleep of child at home (more/less) Usual sleep of parent(s)
Sleep in hospital	Sleep pattern child and parent(s) Sleep during day (more/less) Differences with sleep at home Daytime functioning child/ parent(s) Experiences with prior hospital admissions
Sleep promotion	Examples, ie, bedtime story-telling, reading, nightlamp, soft music
Influence HCP	Care-related routines/other Nurses doctors/other Sleep promotion/hindering by HCP (examples) Recommendations for HCP
Other environmental disrupters	Promoting/ disruptive factors other than by HCP. Light, noise, movement, Other patients/parents in room

Abbreviation: HCP, health care professionals.

### Data Analyses

Consistent with the phenomenological approach, a qualitative thematic analysis was performed following 6 steps:^26^ (1) The audio recordings of all interviews were transcribed verbatim by 2 research assistants and 2 researchers (C.J.v.d.P. and P.B.). The transcriptions were read by the research team (C.J.v.d.P., P.B., and J.M.) several times for familiarization of data. (2) During the first stage of data collection, 2 interviews were coded and analyzed by C.J.v.d.P. and P.B. independently to reach consensus about the final list of codes. After consensus was reached, the remaining interviews were performed by C.J.v.d.P. The coding of these remaining interviews was checked and complemented by P.B. and J.M. (3) By comparing the generated codes with each other and re-reading the associated quotes, categories were formulated. (4) In several meetings with the research group, the categories were critically discussed and adapted, constantly returning to the codes and the transcripts, until (5) consensus was reached on the final themes after which (6) the final research report was written. The data analysis was facilitated by MaxQDA-software.^27^

### Trustworthiness (Validation)

Several techniques were used to increase the trustworthiness of the findings. Trustworthiness was established in accordance with the standard criteria for qualitative research: credibility, dependability, conformability, and transferability.^24^ To enhance credibility, the interviews were conducted in a quiet private environment. Immediately following the interviews, field notes were made describing the circumstances and nonverbal communication. Conducting multiple interviews ensured a sufficient saturation of the caregivers’ views about the sleep of their child. The transcripts and preliminary analyses of the interviews were sent to the participating parents, to confirm that the interpretations accurately reflected their views and experiences (member check). Furthermore, various consensus meetings were held with the research group during the data collection and analyses, moving carefully back and forth between the data and the various steps in the analysis. The analysis was discussed by the authors until agreement was reached.

To increase study dependability, the coding that resulted from the transcripts and the subsequent themes were checked and reconfirmed by 2 researchers that were not involved in data collection (P.B. and J.M.).

To promote conformability and transferability of the findings, extensive descriptions of the selection, data collection, and analysis processes have been described, including context and characteristics of the participants.

Last, all quotes have been translated by one researcher (P.B.) and validated by all the co-authors to ensure correctness and to capture the tone/intonation of the parent(s). Occasional discrepancies were solved by consensus discussions.

### Ethical Considerations

All data were treated according to the European General Data Protection Regulation and were anonymized before analyzing and reporting. All participating parents gave informed consent and permission to make audio recordings. All data were stored on a secured hospital server.

## Results

Thirteen parents were interviewed. The audio recording failed for one interview, and therefore had to be excluded. One of the interviews was conducted with a parent couple. The mean age of their children was 6.9 (range = 1-12) years, and 6 of them were girls. The length of hospital stay varied between 2 and 8 days. Nine admissions were unplanned. The admission diagnosis varied between the children. An overview of the characteristics of the children and parents is presented in Table 2.

**Table 2.** Demographics of Participants.

Parents (N = 12)	
Gender parent	
Female, n (%)	6 (50.0)
Age mother, mean (SD)	39.0 (8.4)
Age father, mean (SD)	40.3 (6.8)
Marital status, n (%)	
Married	10 (83.3)
Divorced	2 (16.7)
Patients (N = 11)	
Gender child	
Female, n (%)	6 (54,5)
Age child, mean (SD)	6.9 (4.1)
Length of stay at interview, mean (SD)	4.3 (2.2)
No of admissions total, n (%)	5.6 (3.0)
No of admissions AUMC, n (%)	3.6 (2.0)
Type for admission, n (%)	
Unplanned	9 (81.8)
Planned	2 (18.2)
Admission diagnosis	
Airway infection Acute myeloid leukemia Surgery for appendicitis Surgical straightening of foot (muscle disease) Epstein-Barr/congenital bronchomalacia Volvulus Baclofen pump placement/cerebral palsy Multi System Inflammatory Syndrome fever/rash Chronic abdominal pain Noro virus—dehydration Respiratory syncytial virus infection	

Abbreviations: AUMC, Amsterdam University Medical Centre; n, number.

### Reduced Sleep Quality During Hospitalization

The first aim of this study was to gain a better understanding of inpatient sleep before focusing on disrupters and promotors of inpatient sleep and the role of health care professionals (second and third aim of this study, respectively).

Nine parents indicated that their child’s sleep quality was reduced in the hospital compared with home. In their opinion, sleep duration, number of awakenings, the ease of falling asleep, and bedtime were affected by the hospitalization:

If I compare it with home: when a child is ill, but without the need for hospitalization, it lays in bed in a quiet room all day. But here [in hospital] it’s a jungle, it is chaotic here. The difference between home and hospital is very big. (Interview 11)

According to all parents, poor sleep quality is explained mainly by the severity of the child’s illness. For example, awakenings were caused by pain, nausea, or vomiting. Depending on the exact time of waking up during the night, children had difficulty falling back to sleep again. Two parents indicated, however, that their child was able to fall asleep quickly again after being disturbed. Furthermore, parents indicated that the number of disturbances at night by health care professionals was associated with the severity of illness:

And at home she also sleeps continuously through the night, whereas here I notice her sleep is disturbed more easily. (Interview 5)

The impact on sleep during admission differed among the children. Some parents indicated that their child was unable to sleep during the day, even when the child was drowsy and tired. Others indicated that their child was exhausted by the poor night’s sleep causing him/her to catch up sleep during daytime. As a result, the child’s fatigue and sleepiness during the day were strongly influenced by the amount of sleep. In addition, daytime functioning seemed also impaired, where some children had no energy to do something fun during the day due to poor nighttime sleep. Most parents did not think poor sleep had an impact on their child’s mood, even though they responded more emotionally due to their illness. Parents said that both sleep quantity and sleep routines were very different in the hospital compared with home. In the hospital, the children usually went to sleep later. Some parents tried to adhere to bedtime routines from home, but often failed due to the different circumstances during hospitalization.

Parents who had been in hospital with their child more often, mentioned that children had to recover at home from the lack of sleep in the hospital. Other parents were confident that their children would return to their normal sleep rhythm after discharge:

I noticed when she [the child] comes home after discharge, she indeed sleeps much longer in the morning than she normally does. So we always say: she needs to refuel. (Interview 2)

### Main Themes

**Figure 1.**
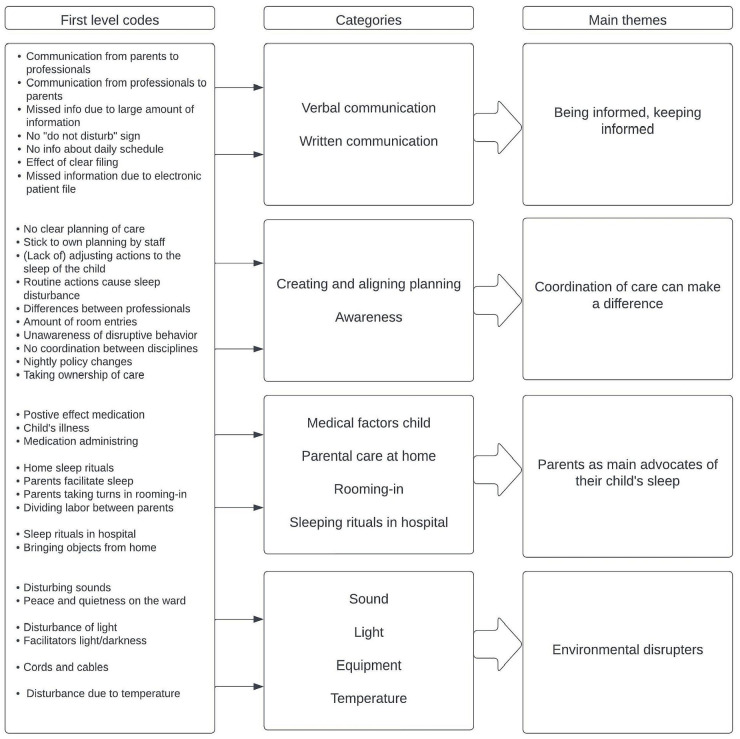
Processing and aggregation of information.

Four main themes emerged from the interviews: (1) being informed, keeping informed; (2) coordination of care can make a difference; (3) parents as main advocate of their child’s sleep; and (4) environmental disturbers (see Figure 1). The themes are separately described below.

#### Being informed, keeping informed

Child’s sleep quality during both night and day appears to be highly dependent on the communication and information exchange between parents and professionals. For example, parents experienced that information provided about their child’s sleeping habits were not transferred among health care professionals. As a result, health care professionals did not always consider the agreements made about the schedule and routines. Parents attributed this to a lack of information exchange between professionals or to information missing in the electronic patient file. Parents suspected that important information was difficult to obtain due to an overload of information.

Parents believed that creating a plan for the upcoming night together with nurses was beneficial for a good night sleep. They appreciated upfront information about the timing of interventions during the night, such as medicine administration. In addition, they also mentioned that dividing care-related tasks and responsibilities among health care professionals and parents was valuable for better sleep quality, for example, the administering of medication:

She [the nurse] said: “I’ll provide it [medication] to you now. If she wakes up, you can give it yourself and then we don’t have to come in; you just let me know at what time you administered it.” . . . That makes it [moment of disturbance] for her as short as possible. (Interview 9)

Parents experienced a lack of information about the child’s program for that day, for example, diagnostic testing, medicine administering. As a result, daytime naps could not be planned:

Of course, . . . you don’t know the schedule. So, you don’t know exactly when things will happen. . . . Like, what time food will arrive, what time breakfast, what time . . . That gives peace. Having a certain routine also gives some peace. (Interview 4)

In addition, health care professionals tend to walk in unannounced and at any time without taking the child’s sleeping rhythm into consideration.

For example, nutrition assistants were often not aware of deviating dietary arrangements. As a result, children were disturbed in their sleep while they were not allowed to eat.

#### Coordination of care can make a difference

Parents experienced a lack of coordination of care, especially during the night. As a result, children were disturbed more often than was necessary according to the parents. Some parents said a good transfer from the evening shift to the night shift had a positive impact on the child’s sleep:

And then during the night, you saw right away when it was well organized. For those moments we didn’t have to remind anyone. In fact, the nurse came in and said: okay, this is how it will happen tonight. Yes, that works very well! (Interview 1)

Parents indicated that they also experienced a lack of coordination between professionals of different disciplines. All professionals seemed to have their own schedule without consulting each other. Consequently, different disciplines entered the patient room scattered throughout the day making it impossible for the child to have moment of rest:

Of course, the doctors only come in when they have time. So, you cannot get that moment clear. And others . . . walk in because you are here anyway. It is difficult to make clear appointments since you do not know how the day will turn out. (Interview 5)

#### Parents as main advocates for their child’s sleep

Parents took the role of guardians for their child’s sleep to ensure that the child would get as much sleep as possible. To ensure that their child feels as safe and comfortable as possible, they ensured that important personal items of their child (cuddly toy, pillow, sleeping bag) are taken from home and tried to continue sleeping routines from home as much as possible in the hospital.

Some parents actively discussed the organization of care with the health care professionals in such a way that their child’s sleep would be supported. For example, some parents decided about the care they could provide themselves on times they considered best, for example, changing diapers. In this way, parents ensured that their child was not unnecessarily disturbed.

Parents mentioned being alert constantly to avoid disturbances they felt were unnecessary. They sometimes closed the curtains at the entrance to the patient room, or put up signs with the message that their child was sleeping. Unfortunately, this did not always give the desired result:

They came in during her sleep to bring food, and another time the cleaner tried to enter although the curtains were closed. So at admission, we asked for a door sign. They gave us a sign that was so beautifully made that no one read it anymore, because it looked like it belonged to the interior. (Interview 1)

Most parents were very understanding and believed disturbances were necessary for their child’s care. However, others were annoyed when their child was disturbed in their sleep but were afraid to speak up on behalf of their child. Parents appreciated it when nurses were advocating for their child’s rest, for example, by negotiating about scheduling medical examinations and making sure other health care professionals did not enter the room during naptime.

Parents took great effort to ensure that the hospital room was low stimulus. For example, they tried to darken the room by putting a towel over the infusion pump and placing the monitor behind the bed curtains. Parents said that they sometimes stayed awake themselves to turn off the alarms of the feeding or infusion pumps quickly, hoping the child would not get disturbed:

As a mother you are always very alert, because you don’t want your child to wake up unnecessarily. So, you press the alarm button quickly, and do not wait for a nurse to come in and turn off that beep. (Interview 2)

Other parents accepted that their child woke up, because the alarms gave them a feeling of security that their child was well looked after.

#### Environmental disturbers

All interviewed parents noticed that their children were exposed to many environmental disturbers. Especially the sounds of monitors, infusion pumps, or feeding pumps made their child wake up, but also lights (power outlets or nurses’ flashlights) were disturbing. Some parents preferred the room temperature to be the same as the child was used to at home and mentioned that room temperature was sometimes adjusted by a nurse without consulting them. Some children were bothered by intravenous lines or monitor cabling, making it difficult to fall asleep or to stay asleep. However, many children woke up briefly and fell back asleep quickly. For some children, going back to sleep was dependent on the timing of the interruption. Contrary to these comments, 2 parents indicated that it was generally quiet and that the lights were dimmed on the ward, outside the patient rooms.

Parents felt that many of these disrupters seemed unavoidable, but that they had an impact on sleep quality:

That thing [infusion pump] will sometimes beep for minutes. Yes, of course I sleep terribly because of that, and she [the child] was also bothered by it. . . . These things are annoying, but because we are in hospital, it cannot be avoided, I think. (Interview 6)

Parents also suggested solutions to several disruptive noises. For example, a mother mentioned another hospital having the drip and feeding pumps outside the patient room. One father indicated that he did not understand that the alarms of the monitor could not be turned off, as these alarms are sent to the nurse’s beeper system anyway.

## Discussion

In this study, the perceived quality of sleep, disrupters and promoters of sleep, and the role of health care professionals in creating a healthy sleep environment for hospitalized children were explored. The results from 12 interviews with parents show that children’s sleep quality is impaired during hospitalization. Four themes emerged from the interviews: (1) being informed, keeping informed; (2) coordination of care can make a difference; (3) parents as main advocates for their child’s sleep; and (4) environmental disrupters.

According to most parents, physical discomfort had a big influence on the inability to fall asleep or to stay asleep during the night. Prior research also reported on the effect of a child’s physical condition on sleep quality.^18,25,28^ Furthermore, physical discomfort could lead to passivity during the day, making it more difficult to fall asleep in the evening.^21^ Other consequences of impaired sleep mentioned were inactivity, no energy to play, being drowsy, or taking more naps during the day. In contrast to previous research,^20^ parents mentioned that mood was not affected by the lack of sleep. In our study, affected mood was attributed to the illness rather than the sleep quality.

Our results show the importance of getting and sharing information about the sleeping habits of the child among the health care professionals, and handing over this information to next shifts. The child’s sleeping habits were not often considered in the working routines. This theme was found in previous studies as well.^19,20,29^ Hospitalized adolescents mentioned feeling uncomfortable due to the unpredictable activities by nurses at night.^19^ The value of a commonly agreed schedule was also highlighted in this study, as adolescents slept better when they knew the schedule for the night upfront.^19^

In our study, parents indicated that a lack of coordination of care is contributing to poor sleep. This is a persistent, yet potentially modifiable finding that is in line with a systematic review among adult patients describing that the extensive network of nurses, doctors, and other health care professionals involved in the care, influenced the sleep quality of patients.^30^

To our knowledge, this is the first study reporting on the importance of parents acting as guardians of their child’s sleep, for example, by reducing the number of interventions during the night, and by creating a low-stimulus environment. Another study reported that adolescents found it reassuring that a parent stayed overnight, but this was attributed to seeking an attachment person.^19^ Parents’ role to advocate for their child’s sleep was not found in previous research. Parents’ feeling they have to act as guardians on behalf of their children could be associated with the lack of information exchange between them and health care professionals.

Environmental factors were considered important disrupters of sleep. Parents mentioned intravenous lines or monitor cables resulting in a limited range of motion and hampering sleep. In addition, major disrupters of sleep were noise and light. These findings are in line with previous research.^17,19,20,25,28,30-33^ However, parents indicated that health care professionals attempted to be quiet and avoid light stimuli, but unfortunately, often with insufficient results.

Parents made recommendations for improvement such as putting a sign on the door. Some parents recommended bundling of care and providing a day schedule on paper, so the child and parents would know what to expect. Previous studies also report on organizational and environmental improvements, such as coordinated staff, daily schedules and the bundling of care, blackout curtains, and better room temperatures.^19,20,25^

### Strengths and Limitations

A strength of this study is the heterogeneous sample of participants of clinically admitted children with their parents. Child’s age varied and sex was equally distributed. In addition, an equal distribution in participation of fathers and mothers was achieved. Another strength of this study was the consideration of researcher bias, where we made sure the interviewer had no treatment relationship with the interviewed parents and their child.

A limitation of this study is the use of proxy reports: The parents were asked about the experiences and perspectives of sleep of the admitted children instead of the children themselves. It is possible that parents’ own sleep quality influenced the perspective on the sleep quality of the child. To reduce this risk, we explicatively asked parents about their sleep quality besides their perspectives of their child’s sleep.

### Conclusions

The sleep quality of children admitted in hospital is substantially less than at home. This may be explained by the child’s physiological or mental state, care-related activities, and environmental factors. Due to inadequate communication and coordination of care, the quality of sleep of the children is regularly compromised. Parents have an important role in facilitating their child’s sleep during admission.

## Recommendations

Children’s sleep in hospitals may be improved by facilitating sleep and embedding sleep-awareness in daily care. The child’s sleep routines should be communicated clearly among all persons involved in the care and documented in the patients’ medical record. To facilitate child’s sleep, we recommend documenting a 24-hour schedule, in communication with child and parents, that includes sleep. Bundling of care should also be a regular topic of discussion. Relatively small interventions could have substantial improvements, such as better communication, limitation of nightly checks, and signs on the door to keep health care professionals out of the room.

Future research should try to collect information about the quality of sleep from (especially young) hospitalized children using measures to assess quality and duration of sleep. In addition, more information is required regarding the factors (environmental, disease-related, care-related, patient-related) that affect inpatient sleep. More knowledge is needed on the consequences of poor sleep quality, for example, on the recovery time of children after hospitalization.

## Author Contributions

CJvdP: Conceptualization, methodology, formal analysis, investigation, writing - original draft, visualization, project administration, validation. PB: Conceptualization, writing – original draft, writing review/editing, methodology, formal analysis, validation, visualization. JM: Conceptualization, methodology, validation, formal analysis, writing - review & editing, validation, supervision. RJBJG: Conceptualization, writing - review & editing, validation, recources, funding acquisition.

## Supplemental Material

sj-docx-1-cpj-10.1177_00099228231188223 – Supplemental material for Parents’ Experiences and Perspectives of Their Child’s Sleep Quality During Hospitalization Supplemental material, sj-docx-1-cpj-10.1177_00099228231188223 for Parents’ Experiences and Perspectives of Their Child’s Sleep Quality During Hospitalization by Cor-Jan van der Perk, Pia Burger, Jolanda Maaskant and Reinoud J. B. J. Gemke in Clinical Pediatrics
