# Supplementary material for: Parents’ Experiences and Perspectives of Their Child’s Sleep Quality During Hospitalization
Source: Clin Pediatr (Phila). 2023 Jul 26;63(6):755–63. doi: 10.1177/00099228231188223 (PMC11103915; doi:10.1177/00099228231188223)
Supplement: sj-docx-1-cpj-10.1177_00099228231188223 – Supplemental material for Parents’ Experiences and Perspectives of Their Child’s Sleep Quality During Hospitalization [file sj-docx-1-cpj-10.1177_00099228231188223.docx]

**Appendix B:** Interview Guide, questions based on topic list

| Questions | Examples of probes |
| --- | --- |
| 1. Would you please describe your child’s sleep at night in hospital? 2. Please tell me something about your child’s experiences with sleeping in the hospital. 3. Could you please describe in what way your child’s sleep differs from home? 4. To what extent does your child's nighttime sleep affect his functioning during the day? 5. (if relevant) What are your child’s experience/ reactions on sleeping with other patients and their parent? 6. Could you tell me something about your own experiences with sleeping in the hospital. 7. Could you please mention factors that influences your child’s sleep and rest at the hospital? 8. Could you give examples of situations that promoted or disrupted sleep at night for your child? 9. In what way does your child have earlier experiences from being admitted to hospital concerning regarding stays in the hospital 10. What things could you or your child do to influence your child’s sleep during the night? 11. How do healthcare professionals affect sleep of your child? 12. Do you have any recommendations for healthcare professionals to better facilitate the sleep of your child? | More or less than usual?  Distribution across the day vs. night?  Differences sleep quality day vs night?  Which care-related routines?  What environmental, disease related factors?  How do you notice that?  What does your child do then? How does he show that?  What happens during the night?  Physicians, nurses.  Are there differences between night and day in care or interactions?  How do they promote sleep?  How do they hinder sleep?  Do you get any advice for the night?  Are there things you wish they could do?  Is there anything you need, what you don’t have now? |
